# Supplementary material for: Transferless Inverted Graphene/Silicon Heterostructures Prepared by Plasma-Enhanced Chemical Vapor Deposition of Amorphous Silicon on CVD Graphene
Source: Nanomaterials (Basel). 2020 Mar 24;10(3):589. doi: 10.3390/nano10030589 (PMC7153506; doi:10.3390/nano10030589)
Supplement: Supplementary file 1 [file nanomaterials-10-00589-s001.pdf]

Supporting Information

Transfer-less inverted graphene/silicon heterostructures prepared by plasma-enhanced chemical vapor deposition of amorphous silicon on CVD graphene

Martin Müller[1], Milan Bouša[2], Zdeňka Hájková[1], Martin Ledinský[1], Antonín Fejfar[1], Karolina Drogowska[2], Martin Kalbáč[2], Otakar Frank[2]

[1] Institute of Physics, Czech Academy of Sciences, Cukrovarnická 10, 162 00 Prague, Czech Republic

[2] J. Heyrovský Institute of Physical Chemistry, Czech Academy of Sciences, Dolejškova 2155/3, 182 23 Prague, Czech Republic

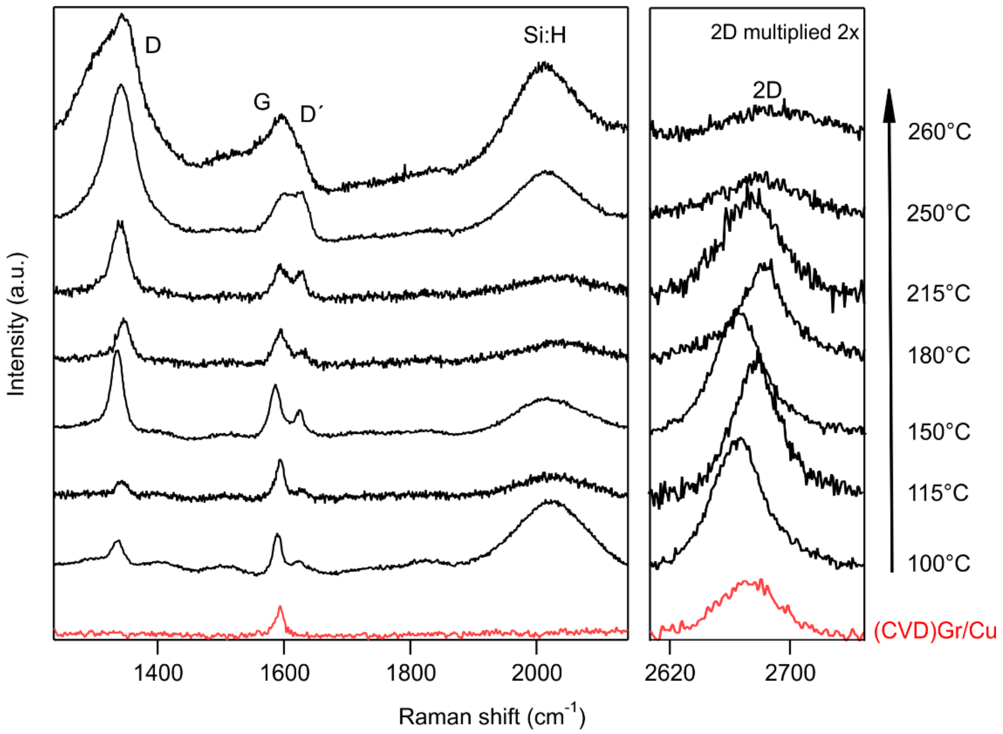

Fig. S1: Single-point Raman spectra of the whole series of the PECVD deposited samples in the range of temperatures 100° – 260°C (black lines) and spectrum of the as-grown graphene lying on the catalytic copper foil (red). The 2D peak region is multiplied 2x for clarity. Different shifts in G and 2D Raman bands position are caused by inhomogeneity throughout the samples (see Fig. S2 and S3). All Raman spectra were measured using 633 nm (1.96 eV) laser excitation wavelength.

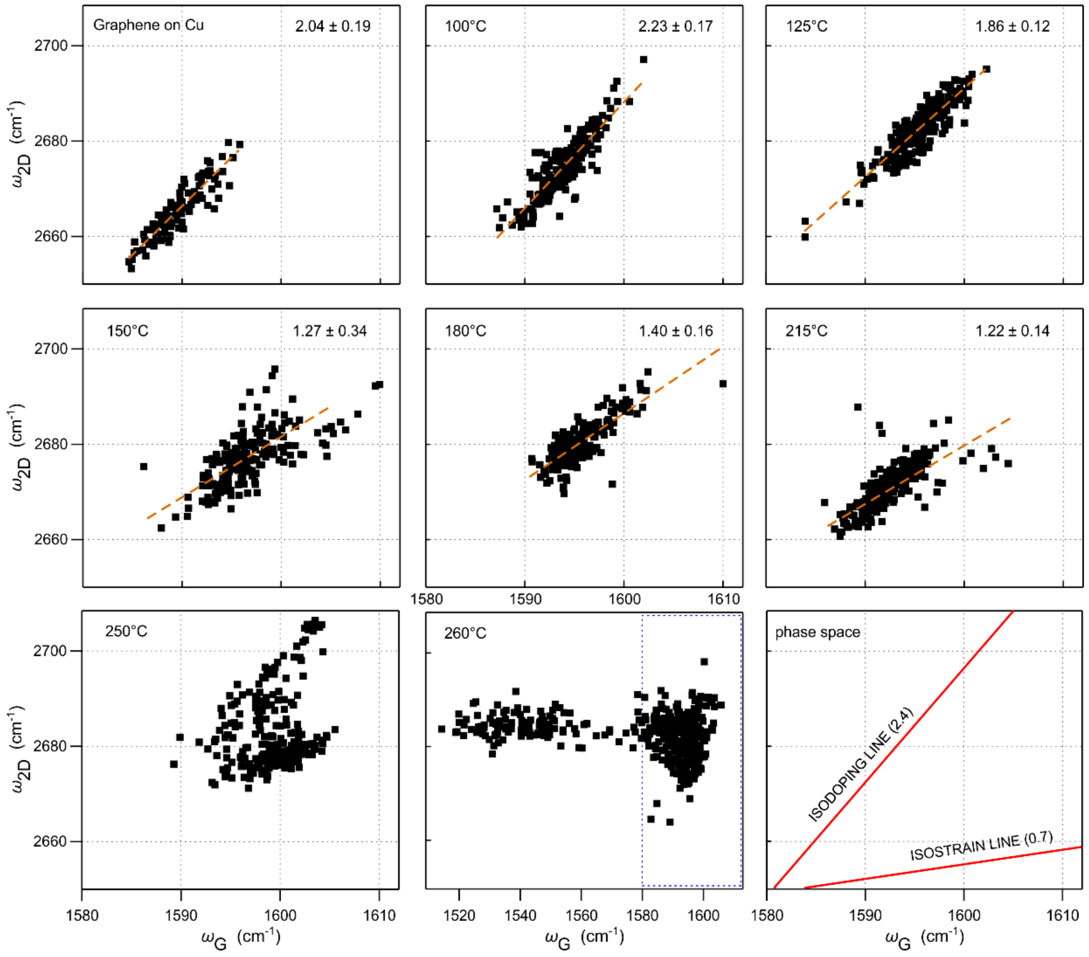

Fig. S2: Correlation plots of G and 2D frequencies acquired by the Raman mapping for the whole temperature series and for as-grown graphene on copper. For every temperature dataset, except for 250° and 260°C, the slope of the line and the line fit (orange dashed lines) are shown. The area marked by the blue dashed-line in 260°C graph is the range of the ω_{2D}-ω_G region depicted in all other plots due to different x-axis range in this case. Additionally, the right bottom panel shows the ω_{2D}, ω_G phase space used for the estimation of doping and strain contribution. The isodoping and isostrain lines show the slope, but neither the absolute positions of the data nor the absolute zero level of the strain/doping.
